# Pulmonary hypertension— a novel phenotypic hypothesis of Kabuki syndrome: a case report and literature review

**DOI:** 10.1186/s12887-023-04273-x

**Published:** 2023-08-28

**Authors:** Xiao-xian Deng, Bo-wen Jin, Shan-shan Li, Hong-mei Zhou, Qun-shan Shen, Yun-yan Li

**Affiliations:** Congenital Heart Disease Center, Wuhan Asia Heart Hospital, 753 Jinghan Road, Wuhan, 430022 China

**Keywords:** Pulmonary hypertension, Kabuki syndrome, *KMT2D* gene mutation

## Abstract

**Background:**

Pediatric pulmonary hypertension (PH) is a serious and rare disease that is often derived from genetic mutations. Kabuki syndrome (KS) is a chromosomal abnormality disease that has its origin in the mutation of lysine methyltransferase 2D(*KMT2D*). Recent evidence has shown that *KMT2D* mutations are associated with pediatric pulmonary disorders. However, the relationship between the clinical courses of PH and the *KMT2D* mutation is reported in extremely few cases. Therefore, in this paper, a case was presented and previous literature was reviewed for better understanding of the correlation between pediatric PH and *KMT2D* mutations.

**Case presentation:**

A 3-year-old girl was transferred to our center for severe cough, shortness of breath, fatigue and fever. Physical examination revealed facial deformities and growth retardation. Echocardiography showed a small atrial septal defect (ASD), and right heart catheterization indicated a significant increase in pulmonary vascular pressure and resistance. The genetic test suggested that she had a KMT2D gene mutation. The patient was finally diagnosed with KS. She was given targeted drugs to reduce pulmonary vascular pressure, but the effect was unsatisfactory.

**Conclusions:**

KS can be complicated with multiple organ malformations and dysfunction. With the progress of next generation sequencing, an increasing number of new phenotypes related to *KMT2D* mutations have been reported. A bold hypothesis is proposed in this article, that is, PH may be a new phenotype associated with *KMT2D* mutations. It is suggested that KS and PH should be differentiated from each other to avoid delayed diagnosis and treatment in clinical practice. There is no specific drug for KS treatment. The prognosis of children with inherited PH is usually poor, and lung transplantation may increase their survival rates.

## Background

Pediatric pulmonary hypertension (PH) is a severe disease with significant morbidity and mortality. The causes of pediatric PH completely differ from those of adult PH [1]. The most frequently encountered types include idiopathic PH, PH due to congenital heart disease (CHD), and lung disease related PH. Kabuki syndrome (KS) is a chromosomal abnormality disease, which has different effects on the development and functions of multiple organ systems. However, there are few reports on pulmonary manifestations of KS patients, especially the correlation between KS and PH. Recent studies have revealed that interstitial lung disease is a new phenotype related to the *KMT2D* mutation of KS [2, 3]. Besides, KS patients have been reported to ultimately die of PH [4]. In this paper, a case of pediatric KS with a *KMT2D* mutation was presented. The patient had manifestations of growth retardation, an atrial septal defect (ASD), congenital hypothyroidism and severe PH. Moreover, it was proposed that PH might be a new phenotype of *KMT2D* mutations.

## Case presentation

**Fig. 1:**
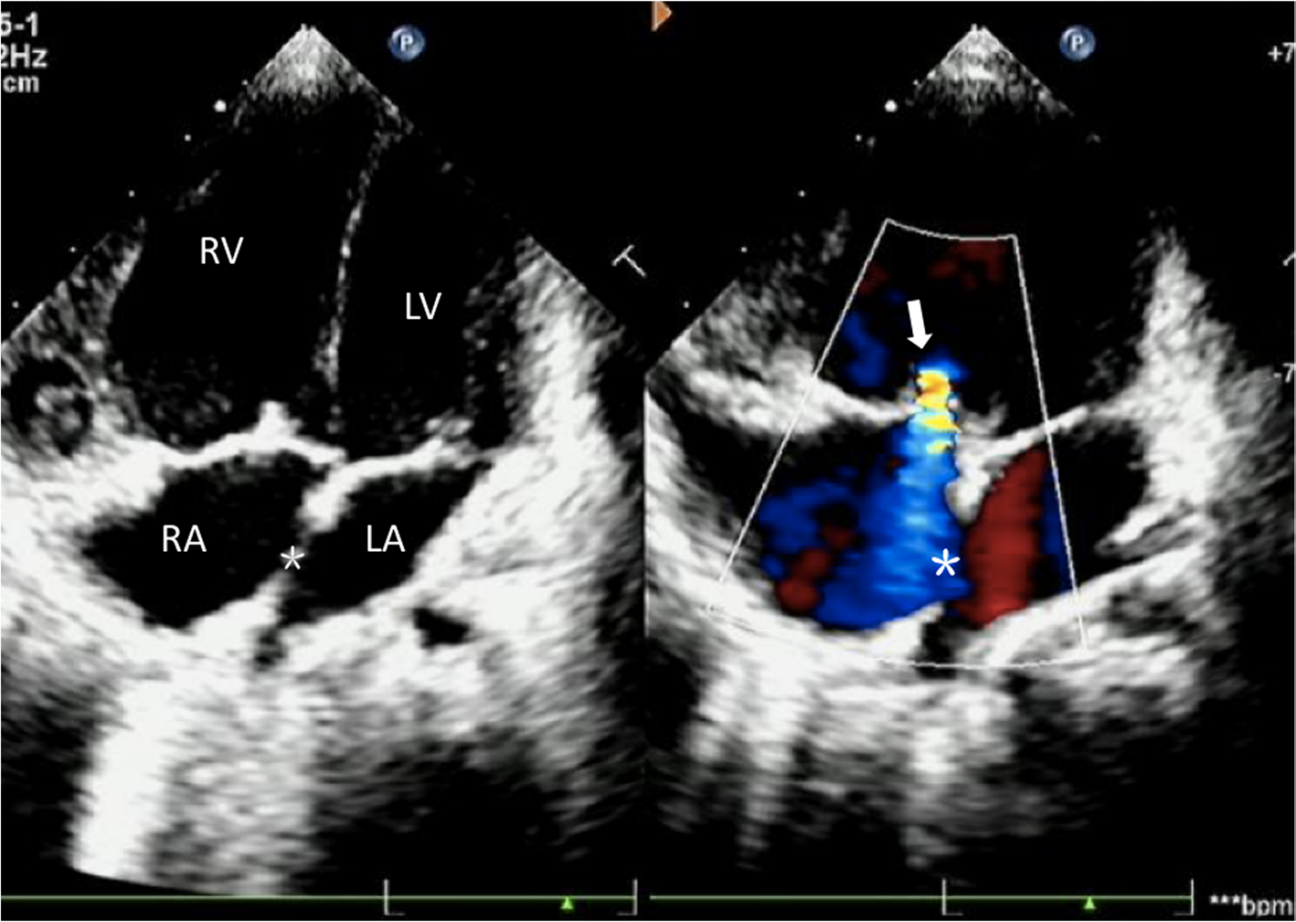
Transthoracic echocardiogram. The four chamber view of cardiac ultrasound shows that the right heart was significantly enlarged,“*”indicates atrial septal defect, the shunt signal of blood flow from right to left can be seen at the defect, "↓" indicates moderate to severe tricuspid regurgitation. LV=left ventricular; LA=left atrial; RV=right ventricular; RA=right atrial

A 3-year-old girl was transferred to our center for severe cough, shortness of breath, fatigue and fever. Physical examination revealed facial deformities (Table 1) and growth retardation. The patient was diagnosed with hypothyroidism, CHD and PH by examinations at the age of 11 months old. Her transcutaneous finger oxygen saturation was 70%. Echocardiography showed a 0.9cm-wide ASD, a widened pulmonary artery, and severe tricuspid regurgitation (Fig. 1). Laboratory tests suggested that NT-proBNP was 3907pg/ml, the C-reaction protein level was 19.83mg/L, and the white blood cell was in the normal range, but the percentage of neutrophils increased to 78.7%, and (respiratory syncytial virus) RSV-IgM was positive. Liver function, kidney function, the level of autoimmune antibodies, and the erythrocyte sedimentation rate were normal. The patient was given anti-infection and cardiotonic drugs as well as respiratory support after admission.

**Table 1:** Summary of the facial deformities of the patient

Organ	Expressions
Eye	Long palpebral fissures; Lateral eversion of the lower eyelids; Arched eyebrows sparse in the lateral half
Ear	Prominent ears; Bilateral asymmetry
Nose	Broad and depressed nasal tip
Mouth	Lower lip concave

**Fig. 2:**
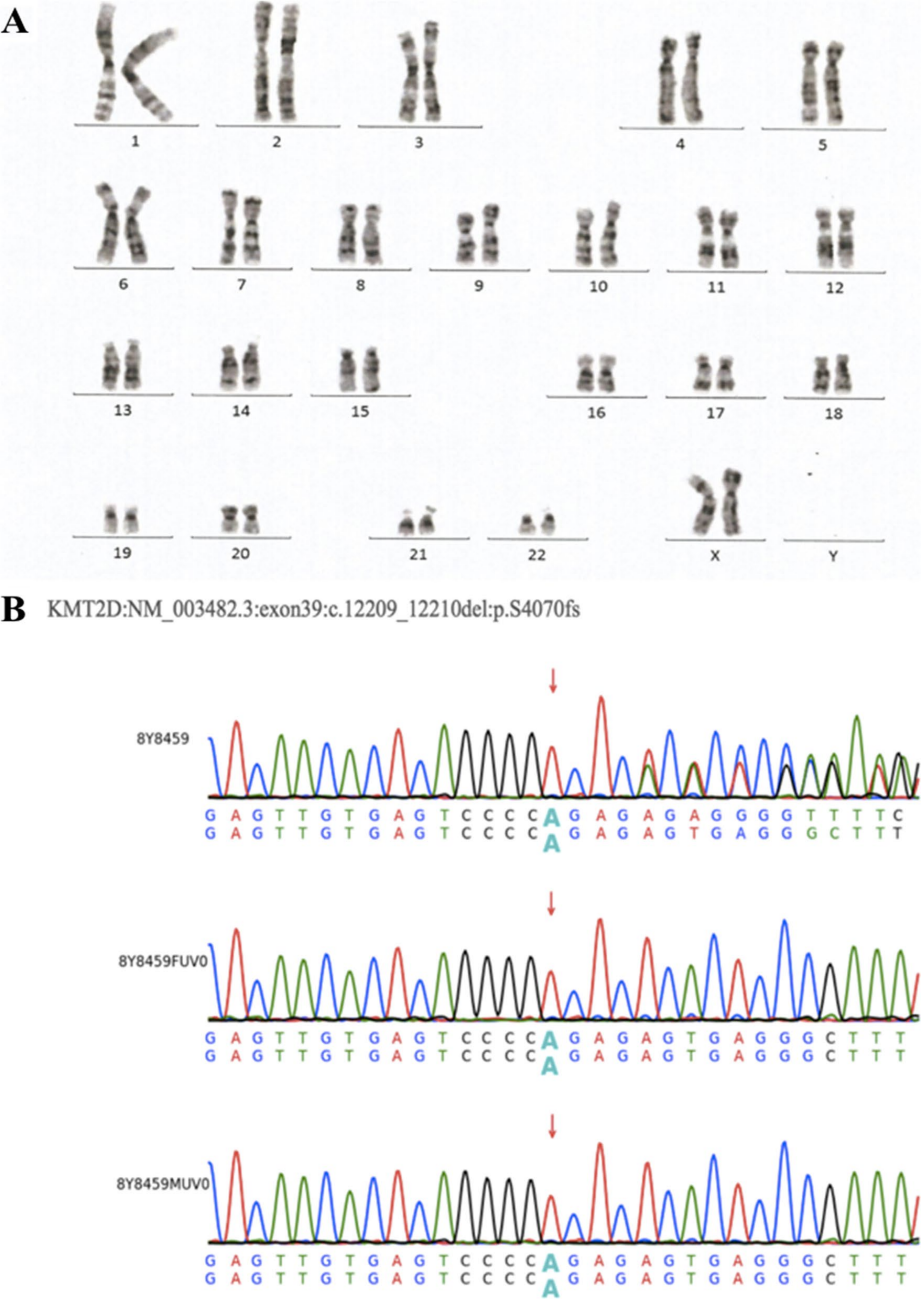
Genetic testing results are as follows. **A **The result of chromosome karyotype analysis shows that: 46, XX [20],which is a normal karyotype. **B** The first generation sequencing verification result shows that the proposiatus had c. 12209_12210del heterozygous mutation on gene *KMT2D*, neither father(the second group) nor mother(the third group) had mutation, which was a new mutation

Meanwhile, the patient underwent right heart catheterization. The results indicated that the mean pulmonary arterial pressure (mPAP, 71mmHg) was markedly increased, the pulmonary vascular resistance (PVR, 27WU) also enlarged, and the pulmonary capillary wedge pressure (PCWP) is normal. Since the ASD was small and the slow flow rate could not result in the tremendous elevation in the pulmonary arterial pressure, the pathogenesis should be further investigated. No obvious abnormalities were found from the results of the pulmonary test, cardiac CTA and other routine etiological examinations in the patient. Nevertheless, the genetic inspection (the accession number for the whole-exome sequencing data is HRA005032) showed that the patient had a *KMT2D (ENSMBL reference ID: NM_003482.3) exon 39 c.12209_12210del p.(Ser4070fs)* mutation (Fig. 2). Based on the genetic results and abnormal countenance, the patient was diagnosed with KS type 1. The patient developed severe PH and poor oxygenation, and she was classified into the high-risk population by risk stratification was. Therefore, she received triple targeted pulmonary vascular pressure reduction treatment, i.e., Ambrisentan (2.5mg once daily) + Tadalafil (10mg once daily) + Remodulin (continuous subcutaneous pumping). The patient was followed up regularly after discharge, but the effect was unsatisfactory. The latest echocardiography reexamination showed no change in the size of the ASD, moderate tricuspid regurgitation, and estimated pulmonary arterial systolic pressure of 96mmHg.

## Discussion and conclusions

PH refers to changes in the pulmonary vascular structure or function caused by multiple etiologies and various pathogens. PH manifests clinical and pathophysiological syndromes due to pulmonary vascular resistance and elevated pulmonary vascular pressure. It can develop into right heart failure and even cause death. There are several factors leading to PH. Idiopathic PH, CHD-PH, and lung disease-related PH are more common in children than in adults. A PH patient can have two or more pathogenic factors simultaneously. To explore the underlying mechanism, the next-generation sequencing is conducted, which reveals genetic defects associated with pediatric PH.

KS is a chromosomal abnormality, which was firstly reported by Japanese scholars Niikawa [5] and Kuroki [6] in 1981. It can cause multiple anomalies, and postnatal growth retardation, hypotonia, and congenital organ malformations are its main clinical manifestations [7, 8]. KS is divided into KS type 1 and KS type 2. KS type 1 exhibits autosomal dominant inheritance [9], and it arises from the mutation of lysine specific methyltransferase 2D*(KMT2D)* (44%~76%) [10–13]. KS type 2 is originated from the mutation of lysine demethyltransferase 6A*(KDM6A)* located on the X chromosome (1%~6%), representing X-linked dominant inheritance [7, 14]. According to some previous literature, *RAP1A* and *RAP1B* gene mutations may be new and rare causes of KS [15]. Other studies have also shown that *KDM6C*, the homologue of *KDM6A*, is another *H3K27* demethylase located on the Y chromosome, and it may act as a candidate gene for KS in male patients [16]. However, early diagnosis of KS remains a challenge in clinical practice since some typical phenotypes of KS only appear with age [17]. High-throughput sequencing technology is applicable to early and accurate diagnosis of KS at the genetic level.

At present, no research has proved a correlation between KS and PH, and the pulmonary phenotype of KS is also rarely discussed. Bang Tami J [3] analyzed CVID-related lung disorders in 2018, and he concluded that KS could cause interstitial lung disease. The conclusion was made based on the fact that KS patients shared similar immune disorders as variable immunodeficiency (CVID) patients. In 2019, Baldridge et al. [2] reported KS cases combined with interstitial lung disease, and they proposed that interstitial lung disease was a new phenotype associated with *KMT2D* variants. Moreover, in a case of KS with respiratory insufficiency, hematoxylin and eosin stained sections of the explanted lung demonstrated extensive alveolar remodeling and septal widening, many pulmonary fibrosis honeycomb structures, and prominent type II pneumocyte hyperplasia. The pathological findings suggested the possibility of PH. In addition, Baldridge also studied a KS patient with decreased physical strength. The echocardiogram of this patient showed elevated right ventricular systolic pressure and moderate tricuspid insufficiency. The electrocardiogram revealed right ventricular enlargement. Although the diagnosis of PH was not confirmed in this patient, the symptoms, echocardiogram and electrocardiogram results were suggestive of PH. In the study of Armstrong Linlea [4], a patient with KS died of PH. Therefore, a bold assumption is proposed in this paper that PH may be a new phenotype associated with *KMT2D* mutations.

There are two possible reasons for the PH in this patient as follows: (1) *KMT2D* gene mutation directly caused the PH, i.e., genetically associated PH(Class I), and for this hypothesis, the PH is considered as a new phenotype of *KMT2D*; (2) A pulmonary disease caused the PH, i.e., pulmonary disease-associated PH(Class III), for it has been reported that *KMT2D* gene mutation may result in pulmonary disease (usually interstitial lung disease). We are more inclined to agree with (1) for the following reasons: (1) Relevant examination for the patient showed no lung-related diseases like the interstitial lung disease or bronchial pulmonary hypoplasia, and currently, there is only PH, and so, it is reasonable to assume that the PH in this patient is genetically related, not associated with a lung disease caused by genetic mutation. (2) Usually, pulmonary disease-related PH is a moderate pulmonary hypertension with moderately elevated pressure and resistance, but the patient's high pulmonary arterial pressure and resistance are severe, which is not consistent with pulmonary disease-related PH. (3) At the same time, targeted drugs for lowering the pulmonary pressure control the progression of PH, which is consistent with the characteristics of Class I PH. Overall, we believe that the PH in this patient is a new phenotype of *KMT2D* mutation.

There is no specific drug for KS treatment. Children with inherited PH often have a poor prognosis. The drugs for preventing or reversing the progression of the disease have unsatisfactory effects. Lung transplantation may increase the survival rate of such patients.

## Data Availability

The raw sequencing data from this study have been deposited in the Genome Sequence Archive in BIG Data Center (https://bigd.big.ac.cn/), Beijing Institute of Genomics(BIG), Chinese Academy of Sciences, under the accession number: HRA005032.

## Abbreviations

PH: Pulmonary hypertension
KS: Kabuki syndrome
*KMT2D*: Lysine methyltransferase 2D
*KDM6A*: Lysine demethyltransferase 6A
CHD: Congenital heart disease
ASD: Atrial septal defect
